# Expression Characteristics of *β-Catenin* in Scallop *Chlamys farreri* Gonads and Its Role as a Potential Upstream Gene of *Dax1* through Canonical Wnt Signalling Pathway Regulating the Spermatogenesis

**DOI:** 10.1371/journal.pone.0115917

**Published:** 2014-12-30

**Authors:** Hailong Li, Zhifeng Zhang, Ying Bi, Dandan Yang, Litao Zhang, Jianguo Liu

**Affiliations:** 1 Key Laboratory of Marine Genetics and Breeding, Ministry of Education, Ocean University of China, Qingdao, China; 2 Yellow Sea Fisheries Research Institute, Chinese Academy of Fishery Sciences, Qingdao, China; University of Washington, United States of America

## Abstract

β-catenin is a key signaling molecule in the canonical Wnt pathway, which is involved in animal development. However, little information has been reported for β-catenin in bivalves. In the present study, we cloned a homolog of *β-catenin* from the scallop *Chlamys farreri* and determined its expression characteristics. The full-length cDNA of *β-catenin* was 3,353 bp, including a 2,511 bp open reading frame that encoded a predicted 836 amino acid protein. Level of the *β-catenin* mRNA increased significantly (*P*<0.05) with *C. farreri* gonadal development and presented a sexually dimorphic expression pattern in the gonads, which was significantly high in ovaries detected by quantitative real-time polymerase chain reaction (qRT-PCR). Immunohistochemical analysis revealed that the β-catenin was mainly located in germ cells of the gonads, with obvious positive immune signals in the oogonia and oocytes of ovaries as well as in the spermatogonia and spermatocytes of testes, implying β-catenin might be involved in the gametogenesis of *C. farreri*. Furthermore, when 0.1 µg/mL and 0.2 µg/mL DKK-1 (an inhibitor of the canonical Wnt pathway) were added *in vitro* to culture medium containing testis cells of *C. farreri*, the expression of *β-catenin* decreased significantly detected by qRT-PCR (*P*<0.05), suggesting the canonical Wnt signal pathway exists in the scallop testis. Similarly, when 50 µM and 100 µM quercetin (an inhibitor of β-catenin) were added *in vitro* to the culture system, *Dax1* expression was significantly down-regulated compared with controls (*P*<0.05), implying the *β-catenin* is an upstream gene of *Dax1* and is involved in the regulation of *C. farreri* spermatogenesis.

## Introduction

The Wnt signaling transduction pathway plays an important role in many developmental processes, including vertebrate limb regeneration, nervous system development, body axis formation, and adrenocortical development [1], [2], [3]. Current studies in invertebrates such as *Drosophila melanogaster*, *Caenorhabditis elegans*, and *Hemicentrotus pulcherrimus*, and vertebrates such as *Danio rerio*, *Xenopus laevis*, *Mus musculus*, and *Homo sapiens* have provided abundant information regarding Wnt signaling in signaling transduction and function [4]–[10].

To date, at least three Wnt intracellular signaling pathways have been identified: the Wnt/β-catenin pathway, the Wnt/planar polarity pathway, and the Wnt/calcium pathway [11], [12], [13]. The Wnt/β-catenin pathway, also named the “canonical” Wnt pathway is the best understood, and controls various developmental processes including cell fate determination, differentiation, and survival, by stabilizing β-catenin [14]. In the absence of a Wnt ligand, cytoplasmic β-catenin is degraded by interactions with a destruction complex formed by three proteins, APC (adenomatous polyposis coli), Axin, and GSK3β (glycogen synthase kinase-3β). The presence of Wnt prevents the degradation of β-catenin, thus the stabilized β-catenin is transported to the nucleus and activates gene transcriptions through direct interactions with T-cell factor/lymphoid enhancer factor (Tcf/Lef) [15]. The Wnt/planar polarity pathway and Wnt/calcium pathway are two major noncanonical Wnt pathways. The Wnt/planar polarity pathway (also called the Wnt/JNK pathway) is characterized by the activation of jun-N-terminal kinase (JNK) involving small GTPases of the Rho family such as RhoA, Rac, or Cdc42 [16]. The Wnt/calcium pathway employs the second-messenger systems of G-proteins to mobilize intracellular calcium stores and activate atypical protein kinase C (PKC) and other calcium-responsive pathways [17], [18]. The noncanonical Wnt pathway is required for the formation of vertebrate tissues, maintenance of adult stem cells, and the suppression of tumors [19].

β-catenin is a key transcriptional effector of the canonical Wnt signal transduction pathway but also functions as a cell adhesion molecule at the plasma membrane by linking cadherins to α-catenin [20]. Structurally, β-catenin consists of an N-terminal region, a central region, and a C-terminal region [21]. The N-terminal region contains a consensus site required for the phosphorylation of GSK-β. The C-terminal region functions as a transactivator required for the activation of target genes [22]. The central region contains 12 imperfect armadillo repeats (ARM). Each repeat forms three alpha-helixes that are arranged in a compact superhelix [23], which is required for interactions with proteins such as cadherins, Axins, APC, and Tcf/Lef [24], [25], [26], [27], [28]. Recently, increasing numbers of studies have found that β-catenin functions in mammalian sex determination and differentiation. In mice, the activation of β-catenin in somatic cells of XY gonads effectively blocks testis development, including disruption of testis cord formation, and down-regulates the expression of testis marker genes, finally leading to male-to-female sex reversal. This implies that mouse β-catenin is a key anti-testis molecule [29]. Furthermore, loss of β-catenin in the SF1-positive population of fetal somatic cells causes morphological defects in ovaries such as the appearance of testis-specific coelomic vessels and the loss of female germ cells, while morphogenesis, Sertoli cell differentiation, or masculinization in testes are not affected, suggesting β-catenin is necessary only for ovarian differentiation but is dispensable for testis development [30]. Moreover, it is well documented in human embryo kidney 293 (HEK293) cells that *β-catenin* activates the expression of *Dax1* through the canonical Wnt pathway. Therefore, the Wnt-β-catenin-Dax1 signaling pathway exists in these cells [31]. Similar results are obtained in mouse ovaries during sexual differentiation, in which the disruption of Wnt4 (an upstream activator of β-catenin) significantly decreased the expression of *Dax1*, implying that this signal pathway may commonly exist in mammals [31].

Shellfish comprise a group of bivalves with rich diversity in species and biological characteristics. Sex is an important characteristic, and sexual variability induced usually by interactions between environmental factors and genes is common in bivalves. However, the molecular mechanisms involved in sex variability are not clear because of a deficiency of data regarding sex-related genes. The scallop *Chlamys farreri* (Jones and Preston 1904) is an important commercial shellfish in China and is characterized by its relatively stable sex composition [32], [33]. Therefore, it is a good experimental species to identify sex-related genes and their functions. In the previous studies, *C. farreri Dax1* is suggested to involve in both oogenesis and spermatogenesis while DAX1 expressed much higher in testis than in ovary [34]. To understand the upstream genes of *Dax1* during *C. farreri* spermatogenesis, we cloned *β-catenin* cDNA in the present study using the Rapid Amplification of cDNA Ends (RACE) technique, and determined its expression pattern in gonads during the reproductive cycle of *C. farreri*. Furthermore, the type of Wnt pathway and correlations between the *β-catenin* and *Dax1* in *C. farreri* spermatogenesis were investigated using an *in vitro* cultured testis cells treated with DKK-1 and quercetin. Our aim was to reveal the β-catenin expression characteristics in the developing gonads of *C. farreri*, and provide evidence for the existence of the Wnt-β-catenin-Dax1 pathway in the *C. farreri* spermatogenesis.

## Materials and Methods

### Ethical statement

The collection and handling of these animals was approved by the Animal Care and Use Committee at the Ocean University of China.

### Animals and sampling

Healthy male and female scallops *C. farreri* with mean shell height 6.27±0.32 cm were purchased from the Aquatic Product Market (Qingdao, China). Gonads were dissected and weighed. Parts of the gonads were immediately frozen in liquid nitrogen, then stored at −80°C until RNA extraction. Parts of the gonads were fixed with Bouin's solution (Picric acid:formaldehyde:acetic acid  =  15∶5∶1) for 24 h, embedded in paraffin wax, sliced at a thickness of 5 µm, and stained by hematoxylin-eosin (H&E) for histological analysis to determine developmental stages of the gonads. Parts of the gonads were fixed in 4% paraformaldehyde (pH 7.4) at 4°C for 24 h, and then dehydrated through a methanol series (25, 50, 75, and 100%) and stored in 100% methanol at −20°C for immunohistochemistry analysis.

According to the morphologic characteristics described by Liao et al. [32], the gonads (ovaries and testes) were grouped into four stages based on histological structure and the gonadosomatic index (GSI  =  gonad weight/soft tissue weight ×100): proliferative stage (GSI = 3.18±0.008 for ovaries and GSI = 3.89±0.008 for testes), growth stage (GSI = 4.20±0.013 for ovaries and GSI = 3.93±0.012 for testes), mature stage (GSI = 4.41±0.004 for ovaries and GSI = 4.53±0.009 for testes), and resting stage (GSI = 2.68±0.006 for ovaries and GSI = 2.46±0.009 for testes).

### Isolation of scallop *β-catenin* full-length cDNA

Total RNA was isolated from *C. farreri* ovaries at the proliferative stage with Trizol reagents (Takara Bio Inc., Otsu, Japan) according to the manufacturer's instructions. The total RNA was then treated with DNase (Takara Bio Inc.) and purified using the RNeasy mini kit (TransGen Bio Inc., Beijing, China). RACE-Ready First-strand cDNA was synthesized, and 5′ and 3′ RACE PCR was conducted using the SMART-RACE cDNA Amplification kit (Invitrogen, Carlsbad, CA, USA). The specific primers (5′-CATTCAGCGTGTAGCAGCAGGAGTCCTC-3′ for 3′ RACE and 5′- CTTCGGACATGCGGAAAAGGACTGCTGC-3′ for 5′ RACE) were designed according to a 325 bp expressed sequence tag (EST) sequence (GenBank accession no. DT718886) with BLAST search from the *C. farreri* EST collection (http://www.ncbi.nlm.nih.gov/nucest/DT718886.1). RACE cDNAs were denatured at 94°C for 5 min, followed by 35 cycles at 94°C for 30 s, 68°C for 30 s, and 72°C for 3 min, ending with a 5-min extension at 72°C. PCR products were separated on an agarose gel (1.2%) and purified with a DNA purification kit (Takara Bio Inc.). Then, the purified 5′ and 3′ RACE products were subcloned into a PMD-18T vector (Takara Bio Inc.) and sequenced.

### Bioinformatics analysis

5′ and 3′ RACE fragments were assembled using DNAstar software (DNAStar, WI, USA) to get the full-length cDNA of *β-catenin*. Sequence identity and similarity of the *C. farreri* β-catenin with other known β-catenins were analyzed using the online BLAST suite of programs at the National Center for Biotechnology Information. Phylogenetic analysis was conducted using MEGA software (version 4.0) with the neighbor-joining method.

### Quantitative real-time polymerase chain reaction (qRT-PCR) analysis

qRT-PCR for analyzing expression levels of the *β-catenin* in gonads of *C. farreri* during the reproductive cycle was conducted as described previously [35]. Total RNA was isolated from the gonads from different stages and was transcribed to cDNA using the PrimeScript RT reagent Kit (Takara Bio Inc.) as the initial templates for qRT-PCR. Specific primers for amplifying a 196 bp fragment were designed according to the non-conservative domain of *C. farreri β-catenin*; forward primer: 5′-CAATCAGCAGCAAGGGTGGA-3′ and reverse primer: 5′-TCTGGGAACATGGCGTCTCG-3′. The *β-actin* gene was used as reference gene following previous studies in *C. farreri* [36], [37], [38], [39] and a 129 bp fragment of *β-actin* (GenBank accession no. AY335441) was amplified using the forward primer: 5′-TTCTTGGGAATGGAATCTGC-3′ and reverse primer: 5′-ATTGTGCTACCACCGGAAAG-3′. Primer specificity during the qRT-PCR was verified by a single distinct peak obtained by melting curve analysis. Quantification of target and reference genes was conducted simultaneously using the ABI 7500 detection system (Applied Biosystems) with SYBR Green Master Mix (Takara Bio Inc.). The qRT-PCR reaction consisted of 5 min at 94°C, followed by 40 cycles of 94°C for 15 s, and 60°C for 1 min. Gonads from six individuals at the same stage were sampled, and duplicate assays for each gonad sample were conducted. Data were analyzed using the ABI 7500 system SDS software version 1.4 (Applied Biosystems) with automatically set baseline and cycle threshold values. The 2^−ΔΔct^ method was used to analyze the relative expression levels of the *β-catenin* [40].

All data were presented as means ± SEM of six samples with two parallel repetitions. Differences between means were tested using one-way analysis of variance (ANOVA) followed by Duncan's post-hoc test (SPSS software version 18.0; SPSS Inc., Chicago, IL, USA), and the significant level was set at *P*<0.05. All assays in the qRT-PCR were validated in compliance with “the MIQE guidelines” [41].

### Immunohistochemistry

Based on the analysis of antigen clusters (DNAstar software), the partial ORF fragment (encoded from 378 amino acids to 836 amino acids) of the *β-catenin* was predicated to have a relative high antigenicity, and was amplified using the forward primer: 5′-GGATCCTCCAGTAACAAGCCAGCTGT-3′ (*Bam*HI site underlined) and reverse primer: 5′-CTCGAGCAGATCAGTGTCATACCAGTTG-3′ (*Xho*I site underlined). After subcloning into a PMD-18T vector (Takara Bio Inc.), the recombinant plasmid was extracted and digested by *Bam*HI and *Xho*I. The digested fragment was then ligated into a *Bam*HI/*Xho*I site of the bacterial expression vector pet28a (Invitrogen) and verified by sequencing. The recombinant plasmid was then transformed into *Escherichia coli* BL21 (DE3) (TransGen Bio Inc.). Glutathione-S-transferase (GST)-β-catenin fusion protein was expressed and affinity purified on an anti-GST Ni-NTA His Bind Resins (Invitrogen), and was injected into New Zealand white rabbits for the production of polyclonal antibodies. Antibody specificity was detected by Western blotting using the gonads of *C. farreri* at the growth stage, as previously described [42]. The sera antibody titer was determined by indirect enzyme-linked immunoassay and the antisera were aliquoted and stored at −80°C. For immunohistochemical analysis, the stored gonads were dehydrated with ethanol, embedded in paraffin, and 5-µm sections were cut using a Histostart 820 Rotary microtome (Reichert, Inc., New York, USA). Sections were deparaffinized in xylene and hydrated in descending ethanol, followed by antigen retrieval in citrate buffer (0.01 mol/L, pH 6.0). To assess the specificity of immunoreactivity, sections of testes and ovaries from different stages were blocked in bovine serum albumin (3%) for 1 h and incubated with pre-immune serum (for the negative control) and anti-β-catenin primary antibody (1∶200 dilution) for 1 h at room temperature, respectively. Specific goat-anti-rabbit IgG (conjugated to horseradish peroxidase, 1∶1,000 dilution) was used as a secondary antibody and incubated for 1 h at room temperature. After washing with PBST (phosphate-buffered saline +0.1% Tween-20), sections were color developed with DAB (3, 3′-diaminobenzidine) and counterstained with hematoxylin. Tissue sections were then observed and images were captured using a Nikon E80i microscope.

### Expression of *β-catenin* and *Dax1* in *in vitro* cultured testis cells treated with DKK-1 and quercetin

Healthy male scallop *C. farreri* with mean shell height 5.67±0.24 cm and mean GSI of 3.95±0.016 from the growth stage were purchased from the Aquatic Product Market (Qingdao, China). The scallops were maintained in seawater for 1 week at room temperature before sampling. The testis tissues were dissected and cut into pieces (mean size  = 1 mm^3^) in PBS (pH 7.4) containing penicillin (1,000 IU/mL) and streptomycin (800 µg/mL). These tissue pieces were cultivated *in vitro* at 23°C in primary medium that consisted of L15 medium (pH 7.2–7.4) plus 5% fetal bovine serum, 20.2 g/L NaCl, 0.54 g/L KCl, 0.60 g/L CaCl_2_, 1 g/L MgSO_4_, 3.9 g/L MgCl_2_, 10 ng/mL EGF, 2 ng/mL bFGF, 10 ng/mL LIF, 100 IU/mL penicillin, and 100 µg/mL streptomycin. After approximately 5 h of adherent culture, most somatic cells and germ cells migrated out from these tissue pieces. According to the differential adherent ability, we made the germ cells apart from bottom of the culture dish and suspend in the medium by shaking slightly the dish, meanwhile the somatic cells and tissue pieces were still attached to the bottom. Then the culture medium containing germ cells was collected carefully and transferred to six-well plates in which new primary medium was added. After cultivating at 23°C for 10 h when the germ cells were completely attached to the bottom of the plate, the culture medium was replaced by new medium containing the new primary medium plus human recombinant DKK-1 (Life Technologies Co., Carlsbad, USA) at the concentrations of 0.1 µg/mL or 0.2 µg/mL, and quercetin (National Pharmacy Inc., Beijing, China) at the concentrations of 50 µM or 100 µM for the treated groups, respectively; for the control, it was added only with new primary medium. The germ cells of the treatment group and control were cultivated for 48 h, and then harvested by centrifugation at 100 g for 10 min. After washing with PBS twice, the germ cells were stored in liquid nitrogen until RNA extraction. Three replicates were conducted for each group. Total RNAs from the cells of each sample were extracted with the MicroElute Total RNA kit (Omega Inc., New York, USA). The RNAs were digested with DNase (Takara Bio Inc.), and transcribed to cDNA as described before. The expressions of *β-catenin* and *Dax1* were detected using qRT-PCR. The primers for *C. farreri β-catenin* were the same as the gonadal expression described above by qRT-PCR detection, and the primers for *C. farreri Dax1* (GenBank accession no. JQ071986) were as follows: forward primer 5′-TCTTCCTCGCCTCATTGTCG-3′ and reverse primer 5′-CGTCGGTATTGGAGCCTTTG-3′. The qRT-PCR data of both treated groups and controls were analyzed by the methods described above.

## Results

### Sequence and evolutionary analysis of β-catenin in *C. farreri*

**Figure 1:**
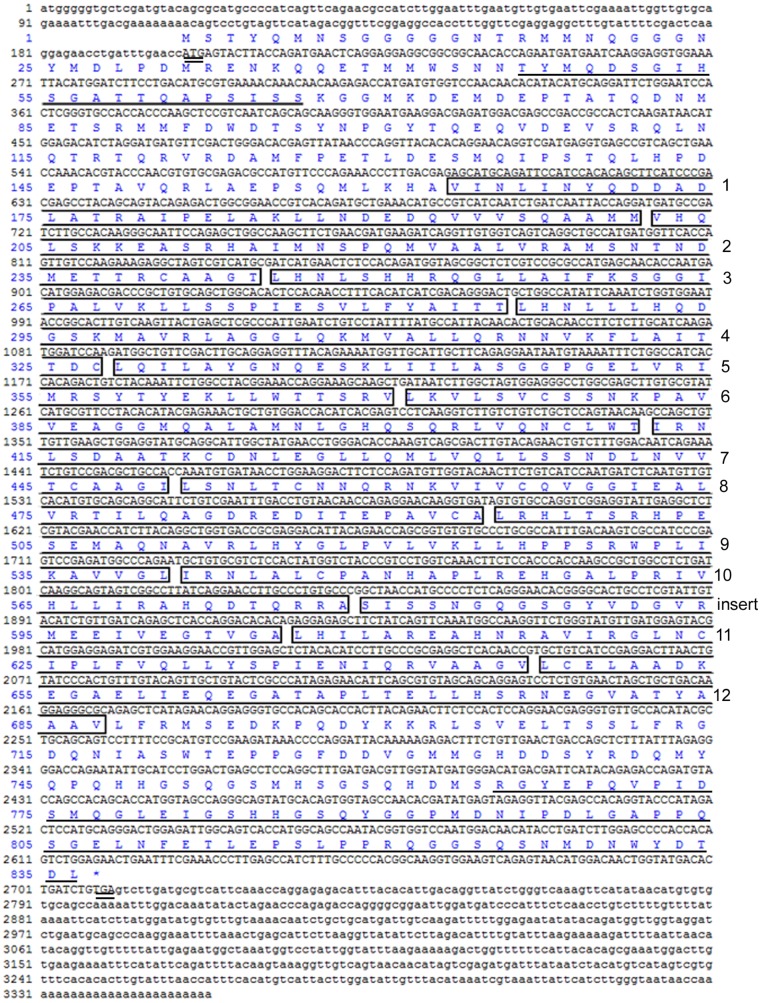
Full-length cDNA sequence and deduced amino acids of *C. farreri β-catenin*. Lower case text indicates the 5′ and 3′ UTR sequences of *β-catenin*; upper case text indicates the encoding sequence. The start codon (ATG) and stop codon (TGA) are double underlined. The putative ARM repeat regions (1–12) are boxed. The N-terminal putative GSK-β consensus phosphorylation site and the C-terminal transactivator region are underlined.

The 3′ and 5′ RACE fragments of target cDNA from *C. farreri* ovary at the proliferative stage were 1,241 bp and 2,279 bp in length, respectively. A full-length sequence of 3,353 bp was assembled (Fig. 1), which included an open reading frame (ORF) of 2,511 bp encoding 836 amino acids, and a 3′ and 5′ untranslated region (UTR) of 643 bp and 199 bp, respectively (GenBank accession number JQ071985). The putative protein was 91.8 kDa with an isoelectric point of 5.76. Structural analysis showed it contained three putative regions of β-catenin: an N-terminal region, a C-terminal region, and a central region. The central region contained a 42-aa ARM in 12 repeats, with a smaller insertion between repeat 10 and 11 (Fig. 1). The N-terminal region had a 21-aa GSK-β consensus phosphorylation site, and the C-terminal region had a 72-aa transactivator domain required for the activation of target gene (Fig. 1). The target protein sequence had high identity with that of other species β-catenin, especially the ARM repeat region that shared 96%, 91%, 89%, 83%, 84%, 83%, 84%, and 84% identities with *Crassostrea gigas*, *Platynereis dumerilii*, *Branchiostoma floridae*, *Strongylocentrotus purpuratus*, *Gallus gallus*, *Ciona intestinalis*, *H. sapiens*, and *X*. *laevis*, respectively. In contrast, the N- and C-terminal regions were more divergent compared with the ARM repeat region. Characteristics described above indicated the target sequence in this study was *C. farreri β-catenin*.

**Figure 2:**
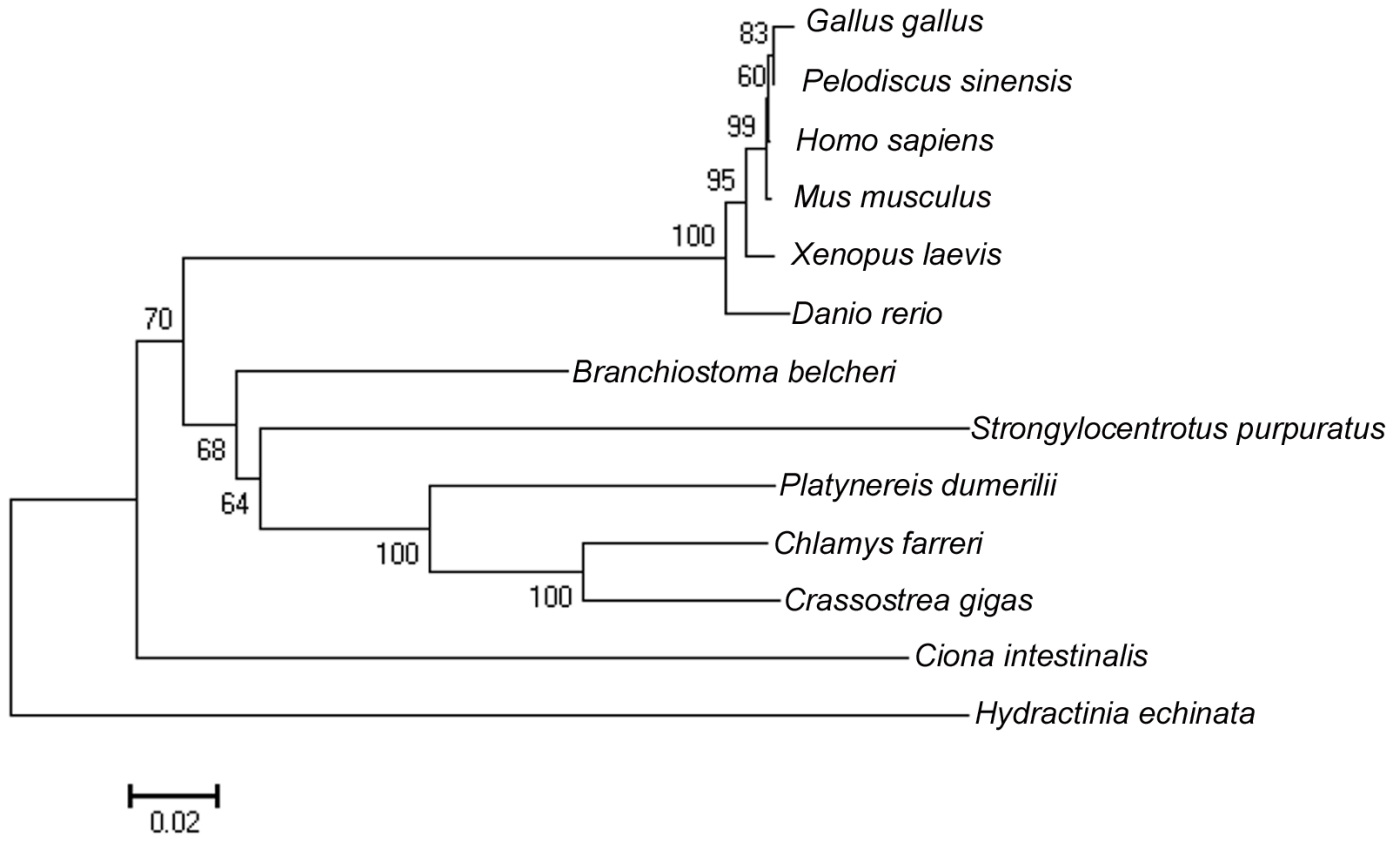
Phylogenic analysis of β-catenin among different species. GenBank accession numbers: *Gallus gallus*: AAB80856; *Pelodiscus sinensis*: BAD74125; *Homo sapiens*: CAA61107; *Mus musculus*: NP_001159374; *Xenopus laevis*: AAA49670; *Danio rerio*: NP_571134; *Branchiostoma belcheri*: BAD12593; *Strongylocentrotus purpuratus*: NP_001027543; *Platynereis dumerilii*: ABQ85061; *Chlamys farreri*: JQ071985; *Crassostrea gigas*: AFL93714; *Ciona intestinalis*: NP_001027779; *Hydractinia echinata*: ACZ56248.

Phylogenic analysis indicated that *C. farreri* β-catenin clustered primarily with that of *C. gigas*, and together formed a subcluster with that of *P. dumerilii*, which then clustered with that of *S. purpuratus* and *Branchiostoma belcheri* successively, finally clustering with the branch formed by vertebrates (Fig. 2).

### Quantitative expression of *β-catenin* mRNA in the gonads during the reproductive cycle

**Figure 3:**
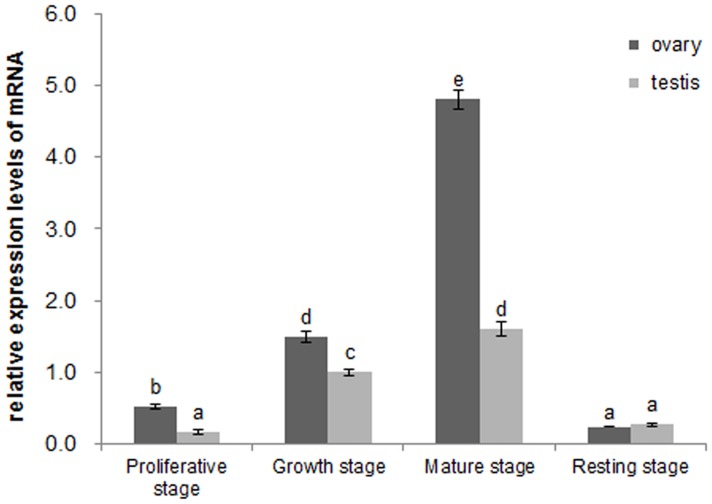
Expression levels of *β-catenin* mRNA in *C. farreri* gonads by qRT-PCR. The expression level of *β-catenin* mRNA in testis at the growth stage is set as 1.00 to calibrate the relative levels in gonads at different stages. Values are the means ± SEM; n = 6. The different letters indicate statistically significant differences (*P*<0.05).

qRT-PCR results (Fig. 3) indicated that the expression of *β-catenin* increased significantly from the proliferative stage to mature stage (*P*<0.05) in *C. farreri* gonads during the reproductive cycle. Then, the level markedly decreased to a minimal level at the resting stage (*P*<0.05). Significant differences at expression levels (*P*<0.05) were also found between testes and ovaries at the same stage except the resting stage, which was approximately two times higher in the ovary than testis at the proliferative stage, 0.5 times higher at the growth stage, and two times higher at the mature stage.

### Cyto-location of β-catenin in the gonads during gametogenesis

**Figure 4:**
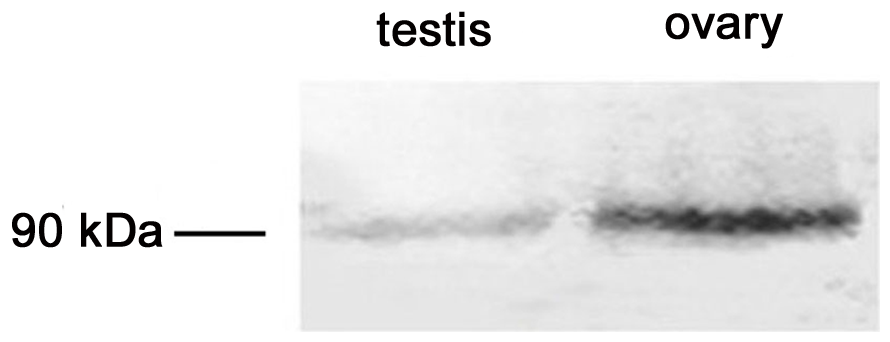
Expression of β-catenin detected by Western blotting in *C. farreri* gonads at the growth stage.

**Figure 5:**
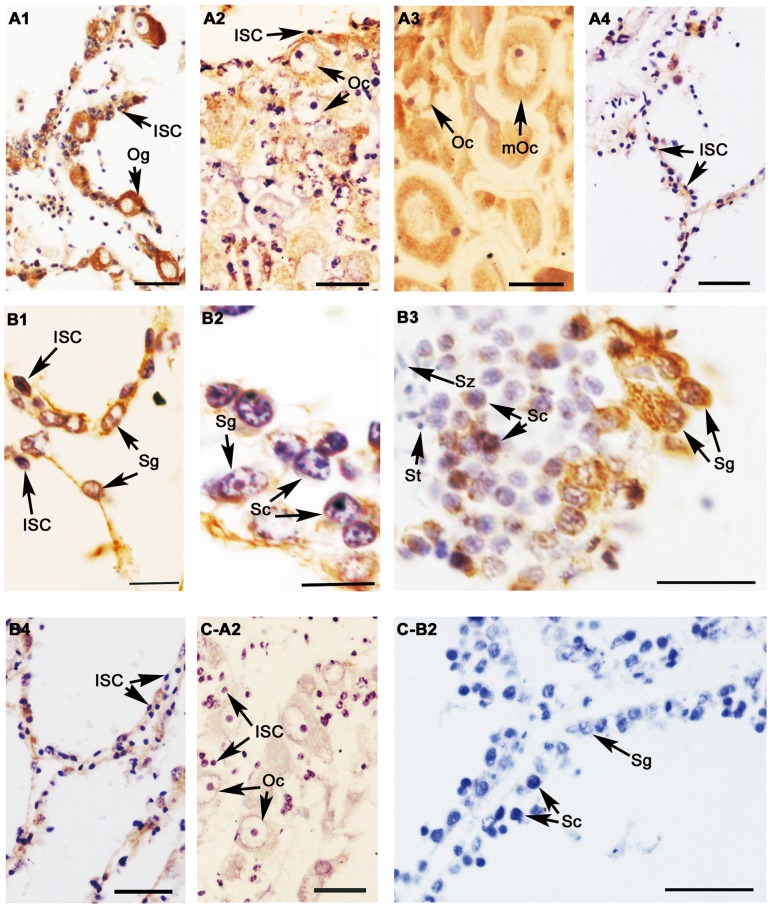
Location of β-catenin protein in *C. farreri* gonads detected by immunohistochemistry. A, B: Positive signals are brown and represent anti-β-catenin in ovaries (A) and testes (B). C: Negative control with preimmune serum; 1: proliferative stage; 2: growth stage; 3: mature stage; 4: resting stage; ISC: intragonadal somatic cell; Og: oogonium; Oc: oocyte; mOc: mature oocyte; Sg: spermatogonium; Sc: spermatocyte; St: spermatid; Sz: spermatozoon; Scale bars: B1, B2, and B3 are 10 µm, others are 30 µm.

The titer of β-catenin polyclonal antibody was detected to be 1∶512,000, and a single band with a molecular mass of approximately 90 kDa in gonads at the growth stage was detected by Western blotting (Fig. 4), indicating the β-catenin polyclonal antibody was specificity. In the testis, β-catenin immunoreactivity was observed in spermatogonia and spermatocytes, however no visible signal was observed in spermatid and spermatozoon during spermatogenesis (Fig. 5 B1–B4). In the ovary, β-catenin was detected in the oogonia and oocytes at all developmental stages (Fig. 5 A1–A4). In testis and ovary of the resting stage, the immunoreactive signals declined to a very low level. Moreover, weak signals were also found in the intragonadal somatic cells (ISCs) which are characterized by small size, extremely heterogenous and pleomorphic nucleus.

### DKK-1 down-regulates *β-catenin* and *Dax1* expression in cultured *C. farrei* testis cells

**Figure 6:**
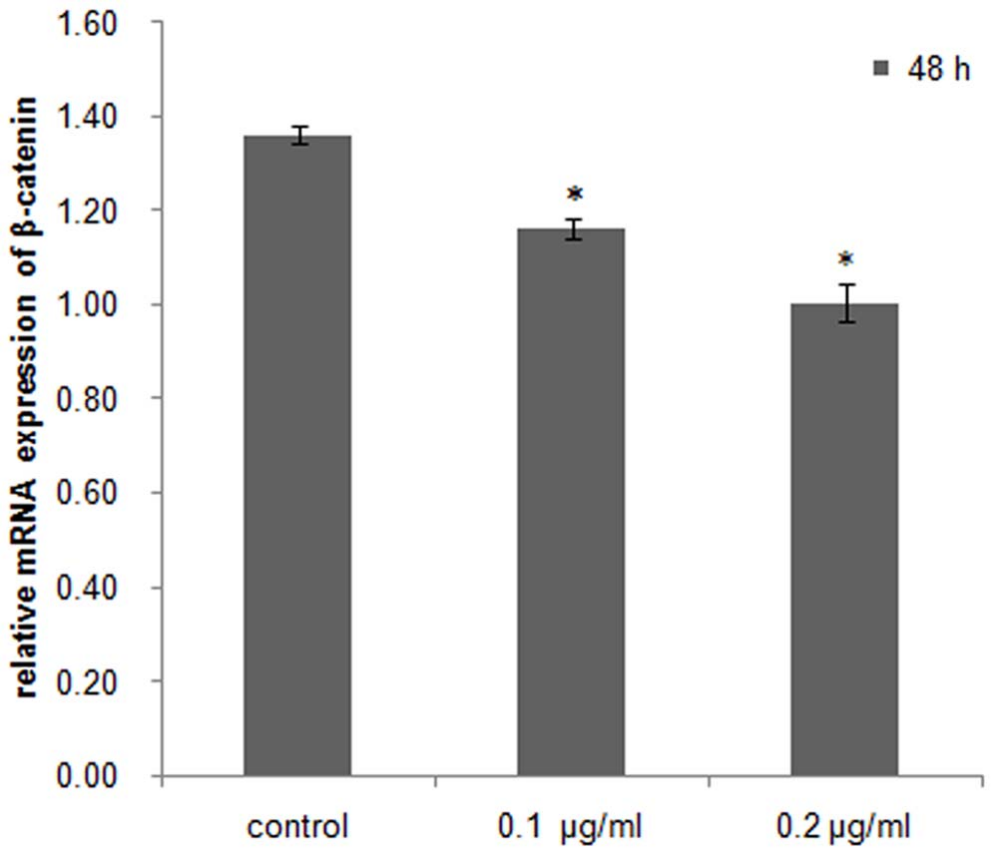
Relative mRNA expression of *β-catenin* in cultured testis cells *in vitro* treated with DKK-1 for 48 h. Asterisks indicate significant differences (*P*<0.05) between the treated group and the control.

**Figure 7:**
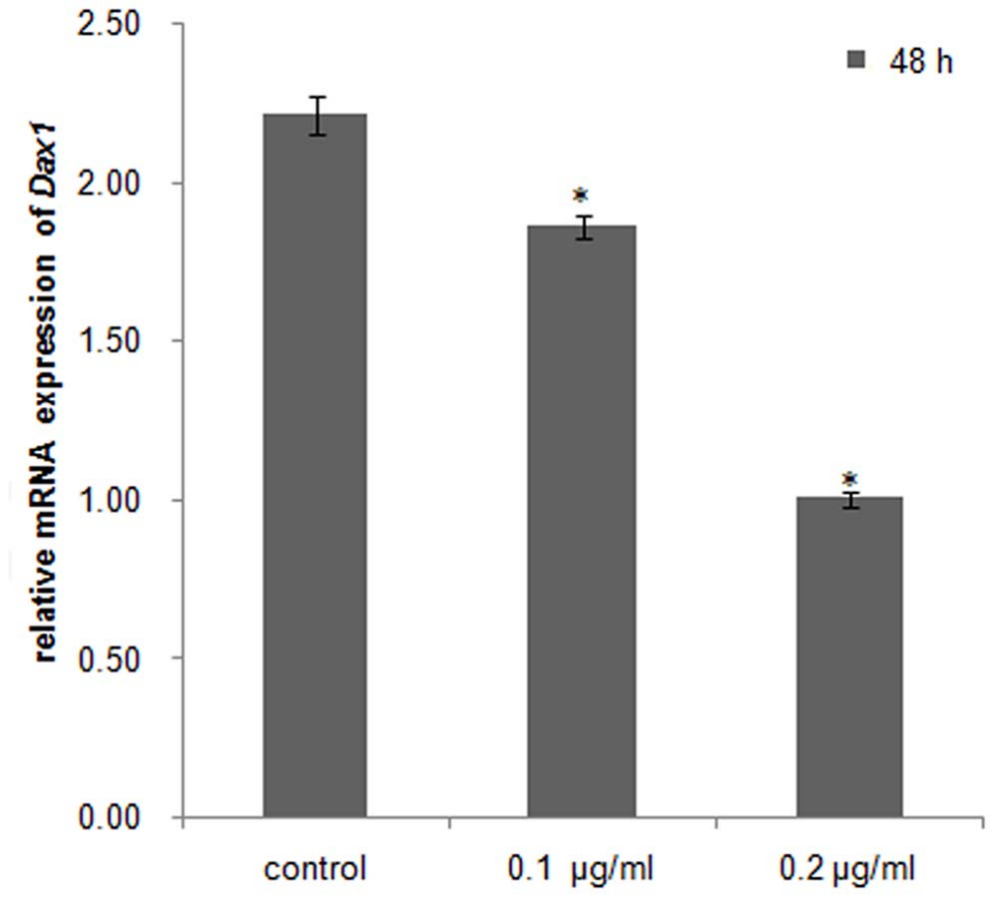
Relative mRNA expression of *Dax1* in cultured testis cells *in vitro* treated with DKK-1 for 48 h. Asterisks indicate significant differences (*P*<0.05) between the treated group and the control.

qRT-PCR analysis demonstrated transcriptional levels of *β-catenin* and *Dax1* decreased significantly (*P*<0.05) in *C. farreri* testis cells cultured *in vitro*, by a concentration-dependent mechanism when DKK-1 was added for 48 h. The expression levels of *β-catenin* decreased about 15% in the 0.1 µg/mL DKK-1 group and 26% in the 0.2 µg/mL DKK-1 group, compared with the control group (Fig. 6). *Dax1* expression levels decreased about 16% and 55%, respectively (Fig. 7).

### Quercetin down-regulates *β-catenin* and *Dax1* expression in cultured *C. farrei* testis cells

**Figure 8:**
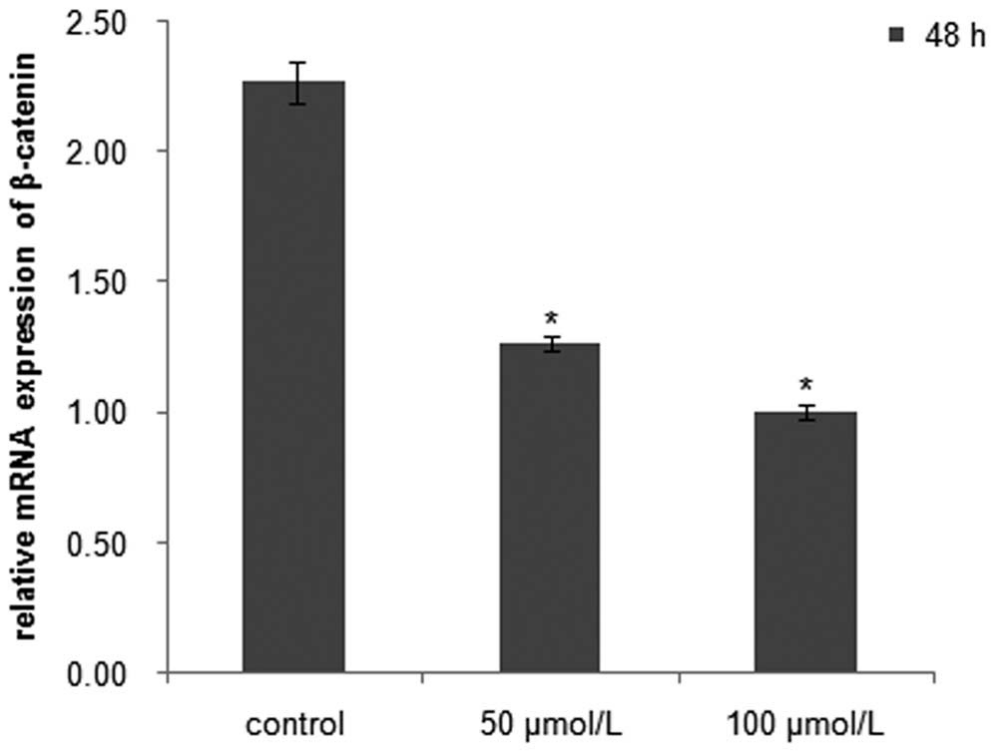
Expression of *β-catenin* in cultured testis cells *in vitro* treated with quercetin for 48 h. Asterisks indicate significant differences (*P*<0.05) between the treated group and the control.

**Figure 9:**
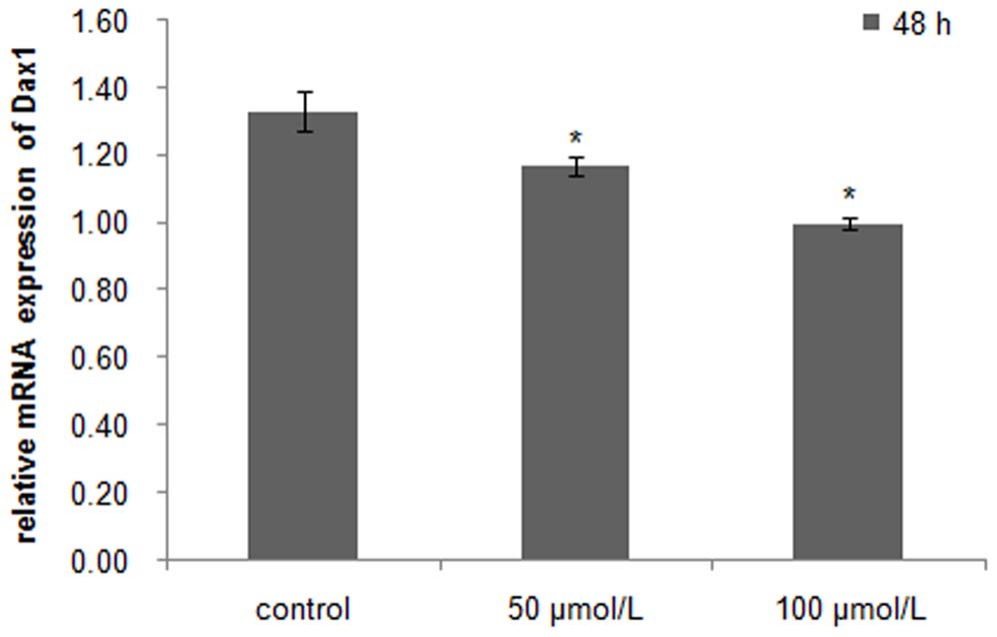
Expression of *Dax1* in cultured testis cells *in vitro* treated with quercetin for 48 h. Asterisks indicate significant differences (*P*<0.05) between the treated group and the control.

Quercetin suppression of *β-catenin* and *Dax1* transcriptional activity in scallop testis cells was concentration dependent. In the cells treated for 48 h, *β-catenin* mRNA levels were reduced by approximately 44% in the 50 µM quercetin group and 56% in the 100 µM quercetin group compared with controls (Fig. 8), and *Dax1* mRNA levels decreased by about 12% and 25%, respectively (Fig. 9).

## Discussion

### β-catenin is an evolutionary conserved protein in *C. farreri*

In the present study, we cloned and characterized a homolog of β-catenin from the scallop *C. farreri*. Like most known β-catenins, *C. farreri* β-catenin has three putative regions, an N-terminal, a C-terminal, and a central region that contains a 42-aa ARM repeating domain that repeats 12 times (Fig. 1). The deduced amino acid sequence of the target protein is highly conservative, particularly in the ARM repeat region shared 96%, 91%, and 89% identities with *C. gigas*, *P. dumerilii*, and *B. floridae* β-catenin, respectively. This result was consistent with previous studies reported in *Hydractinia echinata*, *G. gallus*, *M*. *musculus*, and *D. rerio* [23], [43], [44], [45]. The highly conserved region is thought to be required for β-catenin binding to Axin, a negative regulator of the Wnt signaling pathway, and forming a complex with GSK3β, and interacting with E-cadherin, APC and members of the LEF/TCF family [21]. Most of these interactions are involved in the regulation of the Wnt signaling pathway [22], [46].

### Potential involvement of β-catenin in gametogenesis of *C. farreri*

Generally, the structure and developmental status of the gonads are non-permanent and change as an annual reproductive cycle in most marine animals. In the adult scallop, gametogenesis is initiated annually through mitosis and the differentiation of spermatogonia/oogonia at the beginning of the proliferative stage. This progress is gone on through meiosis and differentiation of germ cells to form mature gametes in the germinal scini of the growth and mature gonads [32]. In the present study, *C. farreri* β-catenin was located in the germ cells (oogonia and oocytes of ovaries, spermatogonia and spermatocytes of testes) and its expression levels increased significantly (*P*<0.05) in both testes and ovaries during the development, implying the β-catenin might be involved in gametogenesis and gonadal development. Furthermore, we observed the expression of *C. farreri β-catenin* presented a sexually dimorphic expression pattern in gonads, with significantly (*P*<0.05) higher levels in ovaries than in testes during the reproductive cycle, except for gonads of the resting stage. Immunohistochemical analysis demonstrated strong β-catenin staining in ovaries while a relatively weak signal was observed in testes, indicating its sex dimorphic expression at the protein level. A similar result has also been observed in mice, where β-catenin is predominately expressed in ovaries [30]. Furthermore, in mouse ovaries, β-catenin is activated by Rspo1, and *Rspo1* knockout mice shows masculinized gonads, suggesting β-catenin is required for the development of ovaries [47]. Based on the similarity of expression, we hypothesized β-catenin might be involved in the gametogenesis of *C. farreri*. Further study is required to reveal the precise function of β-catenin in bivalve gonads.

### The canonical Wnt signaling pathway exists in scallop gonads

Wnt regulates a variety of cellular events via canonical or noncanonical pathways [14], [2]. Wnt antagonists can be divided into two functional classes, the sFRP (secreted Frizzled-related protein) class and the DKK (Dickkopf) class. Members of sFRP include the sFRP family, WIF-1 (Wnt inhibitory factor 1), and Cerberus. The sFRP members can directly bind to Wnts [48], [49] to repress both the canonical and non-canonical Wnt signaling pathways. The DKK class is composed of four members, DKK-1 to DKK-4, which binds directly to the LRP5/LRP6 component of the canonical Wnt receptor complex to specifically inhibit the canonical Wnt pathway [49], [50], [51]. This down-regulates the expression of *β-catenin*, which is a downstream and key mediator molecule in the canonical Wnt pathway [20]. In the present study, the addition of DKK-1 at a concentration of 0.1 or 0.2 µg/mL to cultured testis cells of *C. farreri* significantly (*P*<0.05) down-regulated the expression of *β-catenin* compared with the control. This result is coincident with that of adult human mesenchymal stem cells from bone marrow stroma (hMSCs). In hMSCs, the canonical Wnt signaling pathway has been confirmed to exist, and DKK-1 at a concentration of 0.1 µg/mL can down-regulate the expression of *β-catenin* both in the cytoplasm and nucleus of cultured hMSCs [49]. Thus, our findings in the present study implied that the canonical Wnt signal pathway exists in *C. farreri* gonads.

### *β-catenin* is the upstream gene of *Dax1*

β-catenin is a major component of the canonical Wnt pathway that participates in the regulation of gene expression. In HEK293 cells, β-catenin activated the transcription of *Dax1* by interacting with Ad4BP/SF-1 *in vitro*, thus *β-catenin* is upstream of *Dax1* [31]. The same conclusion is also obtained by an *in vivo* study in mice, where mutation of Wnt4, an upstream activator of *β-catenin*, causes the down-regulated expression of *Dax1*, suggesting the conservation of this signaling pathway in mammals [31]. To identify whether this relationship between β-catenin and Dax1 also exists in *C. farreri*, we examined the expressions of *β-catenin* and *Dax1* in cultured testis cells *in vitro* treated with quercetin, an inhibitor against β-catenin/Tcf signaling that down-regulates the expression of β-catenin or its downstream elements in SW480 colon cancer cells and HEK293 cells [52], [53]. As expected, it was clearly shown in *C. farreri* testis cells cultured *in vitro* that the transcription of *β-catenin* was significantly down-regulated after treatment with quercetin for 48 h, particularly at concentrations of 100 µM (Fig. 8), which down-regulated level by approximately 56% compared with the control. This indicated quercetin inhibited *β-catenin* expression in *C. farreri* testis cells. Furthermore, the expression levels of *Dax1* were also down-regulated when *β-catenin* expression decreased in the cultured testis cells treated with quercetin (Figs. 8 and 9) or DKK-1 (Figs. 6 and 7), suggesting *Dax1* is probably the downstream gene of *β-catenin*.

## Conclusions

In the present study, we cloned and characterized a 3,353 bp full-length cDNA sequence of *β-catenin* from *C. farreri*. The *β-catenin* might play a role in *C. farreri* gametogenesis, and a dimorphic pattern of *β-catenin* expression has been revealed, which was significantly higher in ovaries than testes at the same developmental stage. In addition, we showed the canonical Wnt signaling pathway exists in *C. farreri* spermatogenesis and the *β-catenin* is the upstream gene of *Dax1* in this pathway. The β-catenin in *C. farreri* should be functionally conserved as in mammals.

## Supporting Information

S1 Table **Expression levels of** ***β-catenin*** **mRNA in** ***C. farreri*** **gonads by qRT-PCR.**
(XLSX)

S2 Table **Expression of** ***β-catenin*** **and** ***Dax1*** **in** ***in vitro*** **cultured testis cells treated with DKK-1 and quercetin.** A: Relative mRNA expression of *β-catenin* in cultured testis cells *in vitro* treated with DKK-1 for 48 h; B: Relative mRNA expression of *Dax1* in cultured testis cells *in vitro* treated with DKK-1 for 48 h; C: Expression of *β-catenin* in cultured testis cells *in vitro* treated with quercetin for 48 h; D: Expression of *Dax1* in cultured testis cells *in vitro* treated with quercetin for 48 h.
(XLSX)
